# Emerging V1 neuronal ensembles with enhanced connectivity after associative learning

**DOI:** 10.3389/fnins.2023.1176253

**Published:** 2023-06-29

**Authors:** Yue-Guang Si, Wen-Xin Su, Xing-Dong Chen, Ze-Yu Li, Biao Yan, Jia-Yi Zhang

**Affiliations:** ^1^State Key Laboratory of Medical Neurobiology, MOE Frontiers Center for Brain Science, Institutes of Brain Science, Institute for Medical and Engineering Innovation, Eye & ENT Hospital, Fudan University, Shanghai, China; ^2^Department of Psychology, University of Essex, Colchester, United Kingdom

**Keywords:** associative learning, V1, two-photon calcium imaging, graph theory, ensemble, functional connectivity

## Abstract

**Introduction:**

The visual stimulus-specific responses in the primary visual cortex (V1) undergo plastic changes after associative learning. During the learning process, neuronal ensembles, defined as groups of coactive neurons, are well known to be related to learning and memory. However, it remains unclear what effect learning has on ensembles, and which neuronal subgroups within those ensembles play a key role in associative learning.

**Methods:**

We used two-photon calcium imaging in mice to record the activity of V1 neurons before and after fear conditioning associated with a visual cue (blue light). We first defined neuronal ensembles by thresholding their functional connectivity in response to blue (conditioned) or green (control) light. We defined neurons that existed both before and after conditioning as stable neurons. Neurons which were recruited after conditioning were defined as new neurons. The graph theory-based analysis was performed to quantify the changes in connectivity within ensembles after conditioning.

**Results:**

A significant enhancement in the connectivity strength (the average correlation with other neurons) was observed in the blue ensembles after conditioning. We found that stable neurons within the blue ensembles showed a significantly smaller clustering coefficient (the value represented the degree of interconnectedness among a node's neighbors) after conditioning than they were before conditioning. Additionally, new neurons within the blue ensembles had a larger clustering coefficient, similar relative degree (the value represented the number of functional connections between neurons) and connectivity strength compared to stable neurons in the same ensembles.

**Discussion:**

Overall, our results demonstrated that the plastic changes caused by conditioning occurred in subgroups of neurons in the ensembles. Moreover, new neurons from conditioned ensembles may play a crucial role in memory formation, as they exhibited not only similar connection competence in relative degree and connectivity strength as stable neurons, but also showed a significantly larger clustering coefficient compared to the stable neurons within the same ensembles after conditioning.

## 1. Introduction

Associative learning is a form of learning that enables organisms to understand the relationships among various environmental events and make predictions about the outcomes of their interaction with the environment. In the classic associative learning paradigm, animals are trained to combine neutral sensory stimuli (e.g., visual, auditory, and olfactory stimuli) with an aversive (e.g., foot shock and air puff) or an appetitive event (e.g., sugar water). Experiments that utilized extracellular electrophysiological recordings of single or multiple units found that individual neurons exhibited increased firing rates in response to learning-related stimuli (Segal et al., 1972; Berger et al., 1976; Jun et al., 2017). However, these electrophysiological recordings cannot track the same neuron before and after learning over a long-term period. Intracellular calcium recordings *in vivo* made it possible to track the activity of hundreds of neurons in the same site over days (Hamel et al., 2015). By using this method, Henschke et al. (2020) observed a selective enhancement of the representation in neurons when repeated visual stimuli were associated with a reward. Also, Jurjut et al. (2017) found that learning improved neural discriminability, sharpened orientation tuning, and led to higher contrast sensitivity in the V1 neurons. Recent studies mainly focused on the impact of learning on individual neurons. Considering learning and memory relied on multi-neuronal coactivation, we believe that understanding how these neurons work cooperatively is crucial in exploring the neuronal mechanism for associative learning.

A group of coactivated neurons was typically regarded as ensembles in associative learning (Hebb, 2005; Buzsáki, 2010). Tonegawa et al. (2015) found that activation of the *cfos*-tagged neurons, which was active during the fear conditioning, could induce freezing behavior in mice. Yuste and Bonhoeffer (2001) identified the ensembles in the visual cortex by selecting neuronal pairs with significant similarity from vectors which represented neuronal activity during visual stimulation (Carrillo-Reid et al., 2015) and found that optogenetic activation of these ensembles elicited learning-related behavioral responses such as licking (Carrillo-Reid et al., 2019). They also identified visually evoked and spontaneous ensembles and found that stable neurons in the ensembles were more connected than neurons that were eventually lost in long-term recordings (Pérez-Ortega et al., 2021). These results suggest that stable neurons may play an important role in associative learning. It remained unclear whether new connections were formed in neuronal ensembles after learning.

We combined fear conditioning with *in vivo* two-photon calcium imaging to the same group of neurons before and after fear conditioning. Based on functional connectivity, we identified neuronal ensembles in which neurons were coactivated in response to two different visual stimuli (e.g., conditioned light: blue light; control light: green light), quantified the dynamic changes of the ensembles, and performed graph theory-related analysis on ensembles. Subgroups of neurons were identified as stable, lost, and new ensembles. We found that the new neurons which were recruited in the ensembles after conditioning may play an essential role in associative learning.

## 2. Materials and methods

### 2.1. Animals and surgery

All experimental procedures were approved by the Animal Ethics Committee of School of Basic Medical Sciences at Fudan University. Experiments were performed using wild-type (C57BL/6) male mice (*n* = 8), purchased from the Slac Laboratory Animal Co. (Shanghai, China), and aged 8–12 weeks after birth. The mice were housed in an environment with sufficient water and food. All the animals were maintained on a 12-h/12-h light–dark cycle.

Before surgery, mice were anesthetized with isoflurane (2% for induction and 1% for maintenance). After induction, the scalp was removed and mice were mounted on a stereotaxic apparatus, and three craniotomies (~1 mm in diameter) were made over the right V1 (3.2 mm posterior to bregma and 2.1 mm lateral, 3.2 mm posterior to bregma and 2.5 mm lateral, and 3.6 mm posterior to bregma and 2.3 mm lateral). Then, virus AAV2/8-hSyn-GCaMP6s (Taitool Bioscience Co., LTD, Shanghai, China) was injected using Nanoject (Drummond scientific company, Broomall, USA) at depths of 0.25 and 0.4 mm, respectively. For per injection site, 25 nL of the virus was injected at 20-sec intervals, for a total volume of 100 nL. After each injection, the pipette should be left in place for 5–10 min to prevent backflow. The wound was cleaned and sutured after completing the injections. Then, mice were returned to the home cage.

After 6 weeks, the mice were anesthetized and a 2.5 mm diameter craniotomy was executed above the right V1 (3.3 mm posterior to bregma and 2.3 mm lateral). A coverslip was implanted above the craniotomy and sealed with VetBond (3M Animal Care Products, St. Paul, USA), followed by fixation of the titanium head bar on the skull using dental cement (Super Bond C&B, Japan). After surgery, a dose of (0.1 mg/kg body weight) dexamethasone sodium phosphate (Quanyu Biotechnology Animal Pharmaceutical Co., LTD, Shanghai, China) was injected intramuscularly every other day, and a dose of (5 mg/kg body weight) ceftiofur sodium (Quanyu Biotechnology Animal Pharmaceutical Co., LTD, Shanghai, China) was injected intraperitoneally for 5 days. The mice recovered in the home cage for 2 weeks before two-photon imaging.

### 2.2. Histochemistry

Animals were deeply anesthetized with overdose isoflurane and perfused with 0.9% saline, followed by 4% paraformaldehyde using a perfusion pump. The brains were post-fixed in 4% paraformaldehyde at 4°C overnight and then transferred to 30% sucrose. Brains were then embedded and frozen until sliced. Brain tissues were sectioned into 30-μm-thick coronal slices using a freezing microtome (Leica CM 1950, Leica, Wetzlar, Germany). The slices were washed with Tris-buffered saline 5 times and covered with coverslips. Fluorescent images were obtained by fluorescence imaging microscope (A1R, Nikon, Tokyo, Japan) and analyzed in ImageJ software 1.48v (NIH) and NIS-Elements AR software ver. 4.30.01 (Nikon, Tokyo, Japan).

### 2.3. Fear conditioning

Fear conditioning was carried out using a conditioning chamber (17 × 17 × 25 cm) (Ugo Basile Biological Research Apparatus, Italy) and operated with ANY-maze software (Version 6.33; Stoelting Co., Wood Dale, IL). The software enabled the measurement of freezing response as an indicator of fear memory formation. One day prior to the conditioning session, the mice were placed in the conditioning chamber (context A) and were permitted to explore freely for 300 sec. On the conditioning day, the mice were again placed in context A and explored freely for 180 sec. Subsequently, three blue light stimuli (5 μW/mm^2^) that were co-terminated with a foot shock (0.75 mA, 2 sec) were presented (CS1, CS2, and CS3) followed by a 210-sec inter-trial interval. The freezing level was detected by ANY-maze during the blue light stimulation, and the baseline of the freezing level was calculated during the period of 150–180 sec of exploration. On the day after conditioning, the mice were used for the two-photon imaging experiment.

### 2.4. Two-photon imaging and visual stimulus

A two-photon imaging experiment was performed before and after conditioning. The mice were anesthetized with isoflurane, and the head was fixed in place under a two-photon microscope using a titanium head bar. V1 calcium signal was recorded using Olympus FluoView FVMPE-RS upright two-photon laser-scanning system (Olympus, Tokyo, Japan). The stimulation light was delivered by an LED light source. The LED was positioned 8 cm from the left eye of the mouse, with a light intensity of approximately 5 μW/mm^2^. Blue light and green light were presented six times each in a randomized order, each for a duration of 1 s, followed by a 10-s inter-trial interval. The entire imaging device was enclosed by a blackout fabric to prevent light leakage into the imaging photomultiplier (PMT). The calcium indicator (GCaMP6s) was excited using a laser at a wavelength of 920 nm. Images were acquired at 30 Hz. During the recording, mice were anesthetized with isoflurane (2% for induction and 0.5–1% for maintenance) and placed on a heating pad to maintain body temperature.

For the second recording on the day after conditioning, the location of such target region was attempted to align with the previous area for each mouse. The procedure of the second recording was identical to the first recording.

### 2.5. Pre-process on two-photon image

We use the Suite2p algorithm on python (Pachitariu et al., 2017) to pre-process the two-photon data for analysis. Motion calibration and region of interest (ROI) identification were performed by suite2p on images acquired during recordings. The calcium signal of each ROI was obtained by extracting the fluorescence intensity of the corresponding region. ΔF/F_0_ = (F_raw_ – F_0_)/F_0_, (F_raw_ represents the raw signal of the ROI and F_0_ represents the average intensity of the ROI signal). To improve the signal-to-noise ratio, we applied a smoothing operation to the calcium signal using a window length of 7 frames.

### 2.6. Analysis trials from calcium signal and responsive neuron detection

To analyze the response of neurons, we first aligned the response (ΔF/F0) to the time of stimulus onset. Then, we calculated the average responses with a 2-sec window before the onset (−2 sec from onset to onset) as baseline responses. We also defined an after-stimulus period as a 4 sec window following the stimulus onset. A neuron was considered light-responsive if the maximum ΔF/F0 during the after-stimulus period was more than two times the standard deviation above the baseline responses and the time of decay-half-peak should be over 10 frames in more than 50% of the trials. According to the responses to blue and green light, neurons can be divided into four groups: blue-responsive, green-responsive, both-responsive, and neither-responsive neurons. The proportion of neurons in each group was calculated.

### 2.7. Definition of neuronal ensembles

After obtaining the calcium signal for each neuron, we used a semi-automatic algorithm to identify the neurons in the same location in the two recordings. Pearson's correlation was calculated between each pair of identified neurons within each trial, resulting in a correlation matrix of each trial for all neurons. By averaging all six correlation matrices by the color of stimuli, mean correlation matrices were produced for both blue and green stimuli in recordings before and after conditioning as well. In short, these matrices are named as blue-before-conditioning, green-before-conditioning, blue-after-conditioning, and green-after-conditioning matrices, respectively. A threshold value was settled for each mouse with their ranked 95% correlation coefficient from the blue-before-conditioning and green-before-conditioning matrices. Therefore, pairs of neurons with correlations above the threshold were considered to have functional connections (Bassett and Sporns, 2017). These functional connections represented the synchronous activity between neurons.

After thresholding the averaged correlation matrix and excluding the isolated neurons which have no connections with all other neurons, we performed a clustering analysis by ward linkage based on Euclidean distances on each connectivity matrix. A contrast index was implemented to detect the optimal number of subgroups for clustering (Beggs and Plenz, 2004). The subgroup of the cluster with the highest mean value was selected to represent the ensemble in that condition. For each color of the stimuli, neurons co-existing in the ensembles of both recordings were marked as stable neurons. Neurons that only existed in the before-conditioning ensembles were defined as lost neurons. Neurons that only existed in the after conditioning were defined as new neurons.

### 2.8. Analysis of neuronal network in the ensembles

Neurons in the ensembles and their correlation-based thresholded connectivity were defined as a network of nodes and edges. The binary adjacency matrix which represented all edges between all nodes resulted in an unweighted and undirected graph (Bullmore and Sporns, 2009). Using graph theory-based approaches, both local and global connectivity were assessed. Local connectivity characterized each node of the network, in other words, assessing the connectivity of each neuron in the ensembles. We first investigated the graph measured relative degree. The degree quantifies the number of edges connected to that node (Rubinov and Sporns, 2010). The relative degree is the value of the degree divided by the number of nodes in such networks, therefore, reflects the importance and the strength of such nodes in connectivity within the network. Second, we assessed the clustering coefficient (CC) of each node, which implicated the fraction of the node's neighbors which are also neighbors of each other (Watts and Strogatz, 1998).


(1) $$\begin{array}{lll} CC & = & \;\frac{2t_{i}}{k_{i}\left(k_{i}-1\right)} \end{array}$$

(2) $$\begin{array}{lll} t_{i\;}\; & = & \;\frac{1}{2}\sum_{j,h\in N}a_{ij}a_{ih}a_{jh} \end{array}$$

where *t*_*i*_ represents the number of triangles around a node *i* in the network of *N*, *j h* are neighbors with node *i* and they are also connected, and *k*_*i*_ is the degree of node *i*.

Third, we also determined the strength of connectivity which averaged the correlation value of each node to all other nodes in the ensembles or in the global population (Liu et al., 2019). The connectivity strength reflected the intensity of information transmission between neurons.

Two complementary measures of global connectivity were included in the analysis: global efficiency and small-worldness. The average inverse shortest path length, which is the minimum number of edges required to connect any pair of nodes in a network, is computed as the global efficiency (Latora and Marchiori, 2001), which is implied as a measure of network integration (Achard and Bullmore, 2007). Small-worldness describes the effect of a network on how it clustered than random networks which have similar characteristic path lengths (Watts and Strogatz, 1998). It calculates the ratio of the clustering coefficient and global efficiency in comparison with random networks. The small-world organization is commonly thought on reflecting an optimal balance of functional integration and segregation (Sporns and Honey, 2006).

Neurons in ensembles but had no functional connections with other neurons in the same ensembles were nodes without a degree. Hence, such neurons were excluded in graph theory-based analysis which accounts for the degree.

All network analysis steps were performed in MATLAB using the Brain Connectivity Toolbox (Rubinov and Sporns, 2010) and custom-written scripts.

### 2.9. Statistical analysis

For the statistical analysis, non-parametric tests such as the Mann–Whitney test and Wilcoxon signed-rank test were performed if the data distribution failed to pass the Kolmogorov–Smirnov test; otherwise, parametric tests such as repeated-measures one-way ANOVA, independent sample *t*-test, paired-sample *t*-test, and one sample *t*-test were applied. All tests were performed with GraphPad prime (9.0.0). Group data are expressed as mean ± SEM. The median, quarterlies, and 95% of confidence interval were illustrated in the box charts. The 95% confidence interval and the level of significance (*p* < 0.05) were applied for all analyses.

## 3. Results

### 3.1. The proportion of neurons in mouse V1 responding to conditioned light increased after conditioning

**Figure 1:**
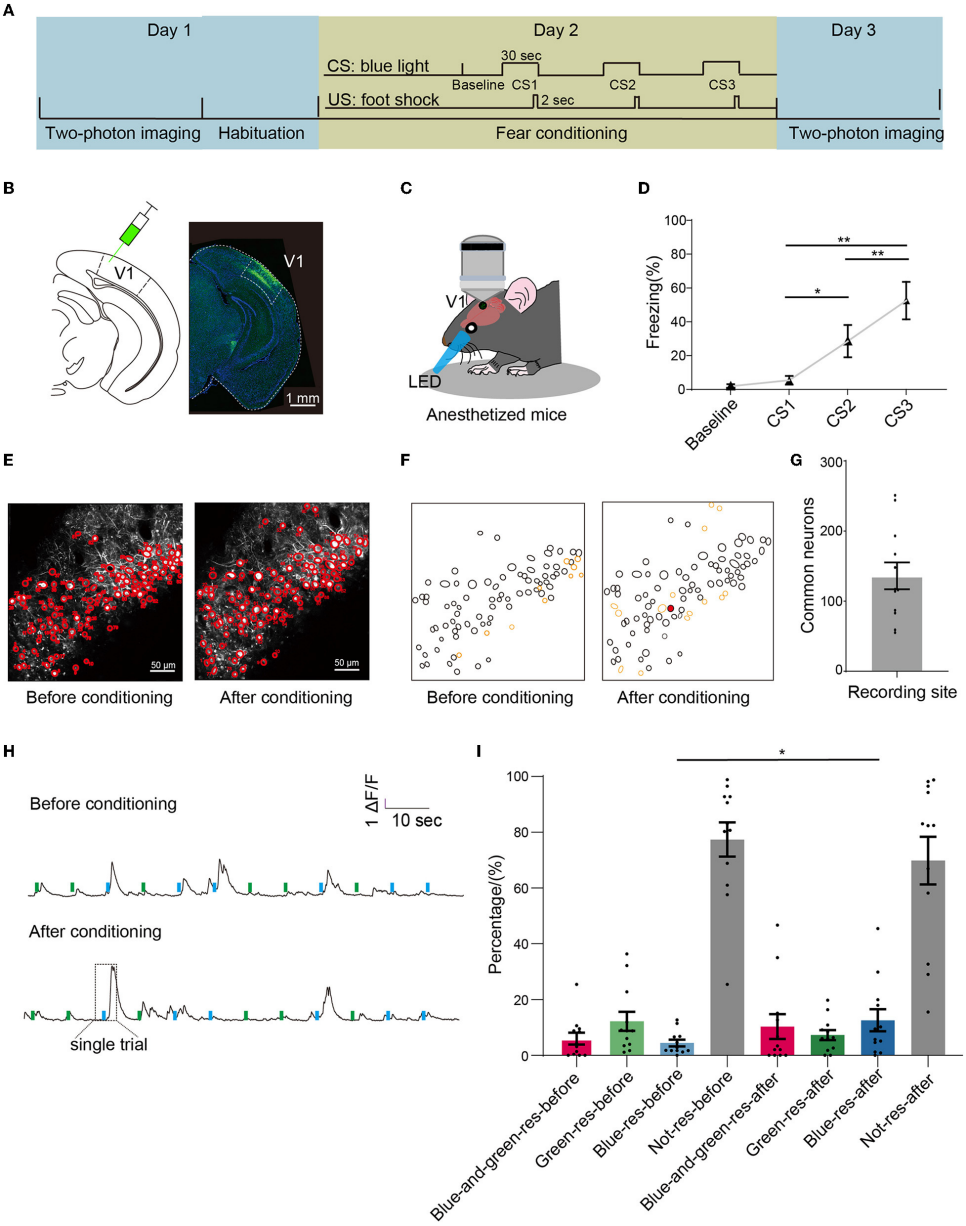
Two-photon imagining before and after conditioning. **(A)** Experimental setup: the first calcium imaging recordings were made 1 day before the fear conditioning of the mice, and the second calcium imaging recording was made 1 day after conditioning. **(B)** Left: schematics of injecting virus AAV2/8-hSyn-GCaMP6s in V1. Right: expression profile of GCaMP6 in V1. Scale bar: 1 mm. **(C)** Schematics of calcium imaging of anesthetized mice given light stimulation. **(D)** Percentage of freezing during conditioning when light stimulation was given three times. Baseline: 2.0 ± 1.2%, CS1: 5.5 ± 2.6%, CS2: 28.6 ± 9.5%, CS3: 52.5 ± 11.1%; one-way repeated measures ANOVA, F = 18.78, *p* < 0.001; Tukey's multiple comparisons tests: *p* = 0.04 in CS1 vs. CS2, *p* = 0.004 in CS1 vs. CS3; *p* = 0.007 in CS2 vs. CS3; *n* = 8 mice. **(E)** Examples of the same recording sites before and after conditioning. Scale bar: 50 μm. **(F)** The common regions of interest (ROIs) based on the Suite2P algorithm in the recording site in E. Black circles represented the ROIs existed in both recording sites; yellow circles represented the ROIs only existed in single recording sites; red circles represented the example ROI in **(H)**. **(G)** The number of common neurons that existed in both Day 1 and Day 3, *n* = 136 ± 19 neurons in each mouse, *n* = 8 mice. **(H)** The original calcium signal of the same neuron was recorded before and after conditioning. Each trail (rectangle with dashed lines) included a 2-sec window before light, a 1 sec window in light, and a 3 sec window after light; each mouse was given six blue light stimuli and six green light stimuli randomly on each day, and the neuron was marked red in **(F)**. **(I)** The percentage of various types of responding neurons before and after conditioning. Blue-responsive neurons before conditioning: 4.4 ± 1.2%, blue-responsive neurons after conditioning: 12.5 ± 4.0%, blue-and-green-responsive neurons before conditioning: 6.0 ± 2.1%, blue-and-green-responsive neurons after conditioning, green-responsive neurons before conditioning: 12.2 ± 3.4%, green-responsive neurons after conditioning: 7.3 ± 1.8%, not-responsive neurons before conditioning: 77.4 ± 6.1%, not-responsive neurons after conditioning: 69.9 ± 8.5%; *n* = 12 recording sites from eight mice for each conditions. Wilcoxon signed-rank test, *p* = 0.027 in blue-responsive neurons before conditioning vs. blue-responsive neurons after conditioning. Mean ± SEM was drawn in the histogram. *p < 0.05, **p < 0.01, and ***p < 0.001.

To investigate the dynamic change in mouse VI neurons after associative learning, we performed two-photon calcium imaging of neurons from V1 layer 2/3 in anesthetized mice before and after fear conditioning (Figure 1A). We injected the virus AAV2/8-hSyn-GCaMP6s into mouse V1 (Figure 1B). 6–8 weeks after the virus injection, we recorded the neuronal activity in response to blue (conditioned light) and green light (control light) on Day 1 (Figure 1C). On Day 2, mice were placed in a conditioning chamber and presented with three pairs of CS-US stimuli. A single trial was consisted of a 30-sec blue light and a 2-sec foot shock terminating simultaneously (with 210-sec inter-trial interval). On Day 3, we recorded the same groups of neurons as those recorded on Day 1 (Figure 1A). The percentage of freezing time evoked by blue light in CS2 and CS3 was significantly larger than that in CS1 (Figure 1D). No significant differences were found between CS1 and the baseline (Figure 1D).

To further explore the effects of associative learning on the plasticity of V1 neurons, we identified the neurons that existed in both Day 1 and Day 3 and defined them as common neurons (Figures 1E–G). We then extracted the calcium signals of individual neurons and analyzed their responses to light (details in method, Figure 1H). According to the responses to blue and green light, neurons can be divided into four groups: blue-responsive, green-responsive, blue-and-green-responsive, and not-responsive neurons. We found that the proportion of blue-responsive neurons increased significantly after conditioning (Figure 1I). No significant differences were found between the proportion of green-responsive, blue-and-green-responsive, and not-responsive neurons before and after conditioning. These results showed that associative learning leads to a selective increase in the proportion of neurons responding to the conditioned light.

### 3.2. Analysis of neuronal populations based on functional connectivity

**Figure 2:**
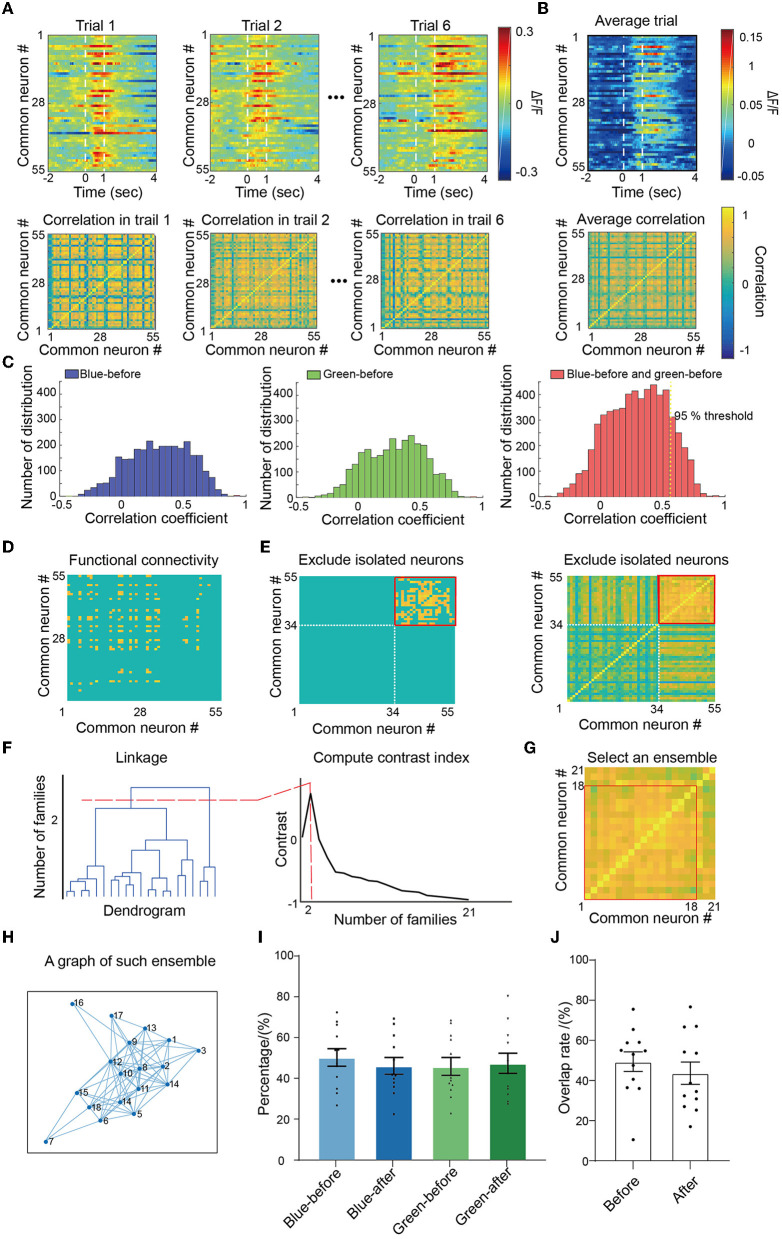
Identification of ensembles. **(A)** For each trial, Pearson's correlation was calculated between each pair of identified neurons and formed correlation matrices within one mouse. **(B)** Average correlation matrices, respectively, for both color and conditions. **(C)** Left: blue-before-conditioning correlation matrix distribution; middle: green-before-conditioning correlation matrix distribution; right: accumulated blue-before-conditioning and green-before-conditioning correlation matrix distribution. A ranked 95% correlation coefficient was picked up as the threshold for functional connection. **(D)** An example of blue-before-conditioning average functional connectivity matrix after applying the threshold. **(E)** Exclude isolated neurons that had no connection with all other neurons. **(F)** Clustering survived neurons with ward linkage and contrast index. **(G)** Find the cluster which has the highest mean correlation value as an ensemble. **(H)** An example graph of such ensemble. **(I)** The proportion of neurons in different ensembles. No significant differences were found in each comparison between pairs of conditions by paired-sample *t*-test; *n* = 12 recording sites from eight mice. **(J)** Overlapping rates between blue-before-conditioning and green-before-conditioning ensembles, and overlapping rates of between blue-after-conditioning and green-after-conditioning ensembles; *n* = 12 recording sites from eight mice. Mean ± SEM was drawn in the histogram.

Owing to the dynamic changes of ensembles in associative learning, we first identified the ensembles in V1 neurons. First, we calculated the correlation coefficient between neurons to construct correlation matrices for each trial (Figure 2A). The averaged correlation matrix, calculated by averaging six trials for each mouse, was used to quantify the connectivity for all four conditions (green-before-conditioning, blue-before-conditioning, green-after-conditioning, and blue-after-conditioning; Figure 2B). We added the distribution of connectivity coefficients for both green-before-conditioning and blue-before-conditioning (red in Figure 2C) and identified the correlation coefficient at the ranked 95% as the unified threshold for all four conditions. As a result, a total of 5% of the connections in blue-before-conditioning and green-before-conditioning were defined as functional connections (connections in blue-before-conditioning are shown in Figure 2D).

We then excluded neurons with no connection with other neurons, i.e., isolated neurons, from the correlation matrix (34 isolated neurons in Figure 2E). To define the coactive neurons (defined as the ensemble in our manuscript), we calculated the distances between neurons with their correlation coefficient. Therefore, a dendrogram was constructed to quantify the linkage between neurons in which we can separate neurons into clusters by their closeness in distance with each other (Figure 2F left). To optimize the number of clusters in such a neuronal population, we calculated the contrast index which compared the inner and outer cluster contrast and picked out the value which suggested the highest differences among clusters (Figure 2F right). Finally, the cluster of neurons that had the largest mean correlation was chosen to be the ensemble for each condition (the chosen cluster formed by 18 neurons in Figure 2G). Hence, we transformed this cluster as a graph in Figure 2H which the neurons were represented as circles and their functional connections were represented as edges.

By following this procedure, we identified all the ensembles associated with blue or green light before and after conditioning. In 48.9 ± 3.8% neurons were in blue-before-conditioning ensemble (blue light-evoked neurons before conditioning), and 49.6 ± 3.4% neurons were in green-before-conditioning ensemble (green light-evoked neurons before conditioning) (Figure 2I). Similarly, 47.4 ± 3.5% neurons were in blue-after-conditioning ensemble (blue light-evoked neurons after conditioning) and 49.3 ± 3.9% neurons were in green-after-conditioning ensemble (green light-evoked neurons after conditioning). No significant difference was found among these four conditions by independent sample *t*-tests. We found a 49.4 ± 4.9% overlapping rate between blue-before-conditioning ensembles and green-before-conditioning and a 43.7 ± 5.6% overlapping rate between blue-after-conditioning ensembles and green-after-conditioning (Figure 2J).

### 3.3. A significant enhancement in connectivity strength in blue ensembles after conditioning

**Figure 3:**
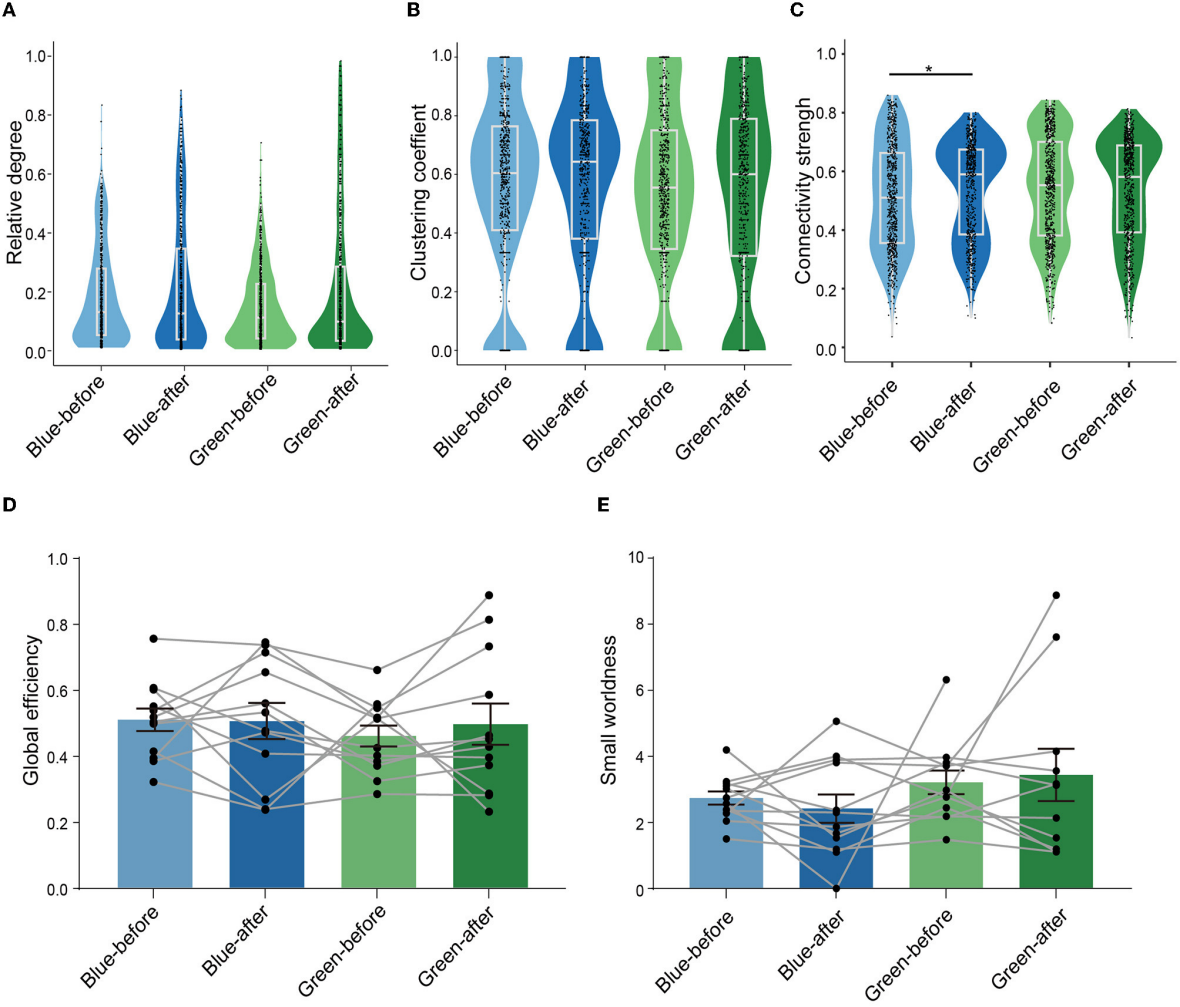
Learning led to enhanced connections of neurons within the ensembles but did not change the configuration of ensembles. **(A–C)** Comparison of the relative degree, clustering coefficient, and connectivity strength of neurons in the four conditions: blue-before-conditioning (*n* = 756), green-before-conditioning (*n* = 791), blue-after-conditioning (*n* = 660), and green-after-conditioning (*n* = 756). Relative degree: neurons in blue-before-conditioning ensembles: 0.185 ± 0.006, neurons in blue-after-conditioning ensembles: 0.217 ± 0.009, neurons in green-before-conditioning ensembles: 0.149 ± 0.005, neurons in green-after-conditioning ensembles: 0.210 ± 0.009. Clustering coefficient: neurons in blue-before-conditioning ensembles: 0.560 ± 0.011, neurons in blue-after-conditioning ensembles: 0.565 ± 0.012; neurons in green-before-conditioning ensembles: 0.535 ± 0.011, neurons in green-after-conditioning ensembles: 0.529 ± 0.012. Connectivity strength: neurons in blue-before-conditioning ensembles: 0.511 ± 0.007, neurons in blue-after-conditioning ensembles: 0.534 ± 0.006; neurons in green-before-conditioning ensembles: 0.538 ± 0.007, neurons in green-after-conditioning ensembles: 0.534 ± 0.006; Mann–Whitney test, *p* = 0.014 in neurons in blue-after-conditioning ensemble vs. neurons in blue-before-conditioning ensemble. **(D, E)** Comparison of global efficiency and small-worldness in the before and after conditions by paired-sample *t*-test in both light stimuli, respectively. Global efficiency: blue-before-conditioning ensembles: 0.509 ± 0.034, blue-after-conditioning ensembles: 0.505 ± 0.054; green-before-conditioning ensembles: 0.460 ± 0.032, green-after-conditioning ensembles: 0.495 ± 0.062; small-worldness: 2.719 ± 0.200, blue-after-conditioning ensembles: 2.403 ± 0.429; green-before-conditioning ensembles: 3.196 ± 0.361, green-after-conditioning ensembles: 3.417 ± 0.791; *n* = 12 recording sites from eight mice. *p < 0.05, **p < 0.01, and ***p < 0.001. Mean ± SEM was drawn in the histogram. The median, quarterlies, and 95% of confidence interval were illustrated in the box charts.

We next performed graph theory-based analysis to quantify the changes in connectivity within ensembles after conditioning. The relative degree (the normalized number of functional connections between neurons), the clustering coefficient (the degree of normalized interconnectedness among a neuron's functionally connected neurons in an ensemble, see formulas 1 and 2 in Methods for definition), and connectivity strength (the averaged correlation value of each neuron to all other neurons in the ensembles) (Newman, 2009) were calculated to quantify local connectivity in the ensembles. The global efficiency (the inverse of the average of the shortest path length between any pair of neurons in the ensemble, a measure of integration which quantifies parallel information transfer in the ensemble, see Methods for details) and small-worldness (a representation of the balance of functional integration and segregation within an ensemble, see Methods for details) were calculated to quantify global connectivity in the ensembles. We found that there was no significant change in relative degree between blue-before-conditioning and blue-after-conditioning ensembles (Figure 3A). Similarly, there was no significant change in relative degree between green-before-conditioning and green-after-conditioning ensembles (Figure 3A). The clustering coefficient remained unchanged after conditioning using either blue or green stimuli (Figure 3B). Interestingly, connectivity strength increased significantly in blue-after-conditioning ensemble comparing to blue-before-conditioning ensemble (Figure 3C).

With regard to global connectivity, no significant difference was found in neither global efficiency (Figure 3D) nor small-worldness after conditioning among all four ensembles (Figure 3E). Further analysis was needed to explore the learning-related plasticity in subgroups of neurons within each ensemble.

### 3.4. Conditioning induced different changes in three neuronal types in the ensembles

**Figure 4:**
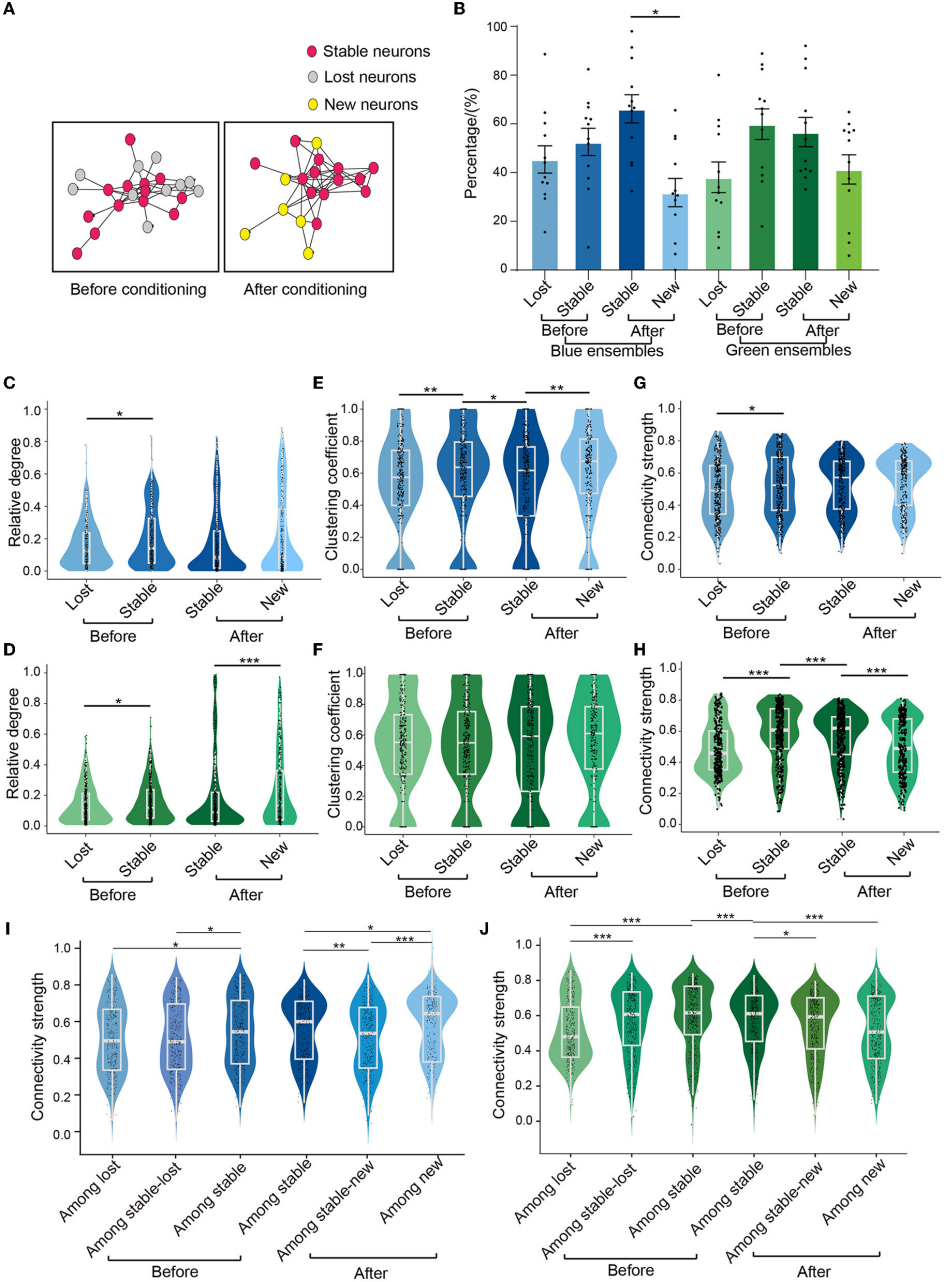
Different neuron subgroups in the ensembles. **(A)** Schematic diagram of different kinds of neurons of an ensemble, red circles represent stable neurons, gray circles represent lost neurons, and yellow circles represent new neurons. **(B)** The proportion of three types of neurons under two colors of light stimulation. Blue condition: lost neurons: 46.3 ± 5.7%, stable-before-conditioning neurons: 53.7 ± 5.7%, new neurons: 32.5 ± 6.0%, stable-after-conditioning neurons: 67.5 ± 6.0%; green condition: lost neurons: 38.9 ± 6.5%, stable-before-conditioning neurons: 61.1 ± 6.5%, stable-after-conditioning neurons: 57.8 ± 6.1%, new neurons: 42.2 ± 6.1%. Paired-sample *t*-test, *p* = 0.012 in new neurons in blue ensembles vs. stable-after-conditioning neurons in blue ensembles, *n* = 12 recording sites from eight mice. **(C, D)** Comparison of the relative degree of three types of neurons in the blue ensembles and green ensembles. In blue ensembles: stable-before-conditioning neurons, 0.204 ± 0.009, *n* = 386; lost neurons, 0.165 ± 0.007, *n* = 370; stable-after-conditioning neurons, 0.196 ± 0.010, *n* = 386; new neurons, 0.247 ± 0.015, *n* = 274; Mann–Whitney test, *p* = 0.004 in stable-before-conditioning neurons vs. lost neurons. In green ensembles: stable-before-conditioning neurons, 0.157 ± 0.006, *n* = 425; lost neurons: 0.139 ± 0.006, *n* = 366; stable-after-conditioning neurons, 0.190 ± 0.012, *n* = 425; new neurons, 0.236 ± 0.014, *n* = 331; Mann–Whitney test, *p* = 0.048 in lost neuron vs. stable-before-conditioning neurons; Mann–Whitney test, *p* = 0.002 in new neurons vs. stable-after-conditioning neurons. **(E, F)** Comparison of the clustering coefficient of three types of neurons in the blue ensembles and green ensembles. In blue ensembles: stable-before-conditioning neurons, 0.583 ± 0.016, *n* = 386; lost neurons, 0.536 ± 0.016, *n* = 370; stable-after-conditioning neurons, 0.532 ± 0.016, *n* = 386; new neurons, 0.611 ± 0.018, *n* = 274; Wilcoxon signed-rank test, *p* = 0.023 in stable-after-conditioning neurons vs. stable-before-conditioning neurons; Mann–Whitney test, *p* = 0.004 in new neurons vs. stable-after-conditioning neurons. In green ensembles: stable-before-conditioning neurons, 0.204 ± 0.009, *n* = 425; lost neurons, 0.165 ± 0.007, *n* = 366; stable-after-conditioning neurons, 0.196 ± 0.010, *n* = 425; new neurons, 0.247 ± 0.015, *n* = 331; **(G, H)**. Comparison of the connectivity strength of three types of neurons in the blue ensembles and green ensembles. In blue ensembles: stable-before-conditioning neurons, 0.527 ± 0.009, *n* = 386; lost neurons, 0.495 ± 0.009, *n* = 370; stable-after-conditioning neurons, 0.525 ± 0.009, *n* = 386; new neurons, 0.547 ± 0.010, *n* = 274; Mann–Whitney test, *p* < 0.001 in stable-before-conditioning neurons and lost neurons. In green ensembles: stable-before-conditioning neurons, 0.580 ± 0.009, *n* = 425; lost neurons, 0.482 ± 0.009, *n* = 366; stable-after-conditioning neurons, 0.563 ± 0.008, *n* = 425; new neurons, 0.497 ± 0.010, *n* = 331; Mann–Whitney test, *p* < 0.001 in stable-before-conditioning neurons vs. lost neurons; Wilcoxon signed-rank test, *p* < 0.001 in stable-after-conditioning neurons vs. stable-before-conditioning neurons; Mann–Whitney test, *p* < 0.001 in new neurons vs. stable-after-conditioning neurons. **(I)** The connectivity strength across subgroups of blue ensembles before and after conditioning. Among lost neurons: 0.504 ± 0.010, *n* = 370; among stable-before-conditioning neurons: 0.541 ± 0.009, *n* = 386; among stable-before-conditioning and lost neurons: 0.509 ± 0.009, *n* = 386; one-way repeated measures ANOVA, F = 4.477, *p* = 0.012; Tukey's multiple comparisons tests: *p* = 0.017 in connectivity strength among lost neurons vs. connectivity strength among stable-before-conditioning neurons, *p* = 0.045 in connectivity strength among stable-before-conditioning and lost neurons vs. connectivity strength among stable-before-conditioning neurons. Among stable-after-conditioning neurons: 0.541 ± 0.009, *n* = 386; among stable-after-conditioning and new neurons: 0.509 ± 0.010, *n* = 362; among new neurons: 0.589 ± 0.010, *n* = 274. One-way repeated measures ANOVA, F = 17.25, *p* < 0.001; Tukey's multiple comparisons tests: *p* = 0.001 in connectivity strength among stable-after-conditioning neurons vs. connectivity strength among stable-after-conditioning and new neurons, *p* = 0.025 in connectivity strength among stable-after-conditioning neurons vs. connectivity strength among new neurons, *p* < 0.001 in connectivity strength among stable-after-conditioning and new neurons vs. connectivity strength among new neurons. **(J)** The connectivity strength across subgroups of green ensembles before and after conditioning. Among lost neurons: 0.507 ± 0.009, *n* = 366; among stable-before-conditioning neurons: 0.594 ± 0.009, *n* = 425; among stable-before-conditioning and lost neurons: 0.567 ± 0.009, *n* = 425; one-way repeated measures ANOVA, F = 23.63, *p* < 0.001; Tukey's multiple comparisons tests: *p* < 0.001 in connectivity strength among lost neurons vs. connectivity strength among stable-before-conditioning and lost neurons, *p* < 0.001 in connectivity strength among lost neurons vs. connectivity strength among stable-before-conditioning neurons. Among stable-after-conditioning neurons: 0.575 ± 0.008, *n* = 425, among stable-after-conditioning and new neurons: 0.544 ± 0.009, *n* = 425; among new neurons: 0.517 ± 0.010, *n* = 331. One-way repeated measures ANOVA, F = 10.30, *p* < 0.001; Tukey's multiple comparisons tests: *p* = 0.027 in connectivity strength among stable-after-conditioning neurons vs. connectivity strength among stable-after-conditioning and new neurons, *p* < 0.001 in connectivity strength stable-after-conditioning neurons vs. connectivity strength among new neurons. Wilcoxon signed-rank test, *p* < 0.001 in connectivity strength among stable-after-conditioning neurons vs. connectivity strength among stable-before-conditioning neurons. Mean ± SEM was drawn in the histogram. The median, quarterlies, and 95% of confidence interval are illustrated in the box charts. *p < 0.05, **p < 0.01, and ***p < 0.001.

We defined neurons that existed in both before-conditioning and after-conditioning ensembles as stable neurons. Stable neurons in before-conditioning and after-conditioning ensembles were defined as stable-before-conditioning and stable-after-conditioning neurons, respectively. Neurons in before-conditioning ensembles that were not present in after-conditioning ensembles were defined as lost neurons, whereas neurons in after-conditioning ensembles that were not present in before-conditioning ensembles were defined as new neurons (Figure 4A).

In before-conditioning ensembles, we did not find the significant difference in the fraction of stable and lost neurons using either blue or green condition (Figure 4B). In after-conditioning ensembles, the fraction of new neurons was significantly smaller than that of stable-after-conditioning neurons in blue ensembles. However, no difference was found between the fraction of new and stable-after-conditioning neurons in green ensembles. These results suggested that fear conditioning altered the constitution change of stable, new, and lost neurons in the blue ensembles in comparison with green ensembles.

Relative degree, clustering coefficient, and connectivity strength were calculated to evaluate local connectivity among four distinct groups of neurons (lost, stable-before-conditioning, stable-after-conditioning, and new). In the blue ensembles, the stable-before-conditioning neurons had larger relative degree than the lost neurons (Figure 4C). No significant difference was found either between stable-before-conditioning to stable-after-conditioning neurons or compared between stable-after-conditioning neurons and new neurons. In the green ensembles, the stable-before-conditioning neurons had larger relative degree than the lost neurons (Figure 4D). No significant result was found between stable-before-conditioning and stable-after-conditioning neurons. Additionally, new neurons had a larger relative degree than stable-after neurons.

Our analysis further revealed that in blue ensembles, stable-before-conditioning neurons exhibited a significant larger clustering coefficient compared to lost neurons (Figure 4E). However, a decline of the clustering coefficient occurred after conditioning for stable neurons. Conversely, new neurons displayed a higher clustering coefficient than stable-after-conditioning neurons. On the contrary, no significant differences in clustering coefficient were found among lost, stable-before-conditioning, stable-after-conditioning, and new neurons in the green ensembles (Figure 4F).

In both blue and green ensembles, the connectivity strength was significantly larger in stable-before-conditioning neurons than in lost neurons (Figure 4G). In the green ensembles, a significantly smaller connectivity strength in stable-after-conditioning neurons than in stable-before-conditioning neurons was observed (Figure 4H). Additionally, new neurons were even weaker than stable-after-conditioning neurons in the connectivity strength (Figure 4H). In the blue ensembles, there was no significant difference between stable-before-conditioning and stable-after-conditioning neurons (Figure 4G). New neurons in blue ensembles had similar connectivity strength compared to stable-after-conditioning neurons (Figure 4G).

We also calculated the connectivity strength among lost, stable-before-conditioning, stable-after-conditioning, and new neurons in blue and green ensembles, respectively. In the blue ensembles, the connectivity strength among lost neurons was significantly smaller than that among stable-before-conditioning neurons (Figure 4I). Similarly, the connectivity strength among lost and stable-before-conditioning neurons was also significantly smaller than that among stable-before-conditioning neurons. The connectivity strength among stable-before-conditioning neurons and connectivity strength among stable-after-conditioning neurons showed no significant change. After conditioning, the connectivity strength among new neurons was significantly higher than that among stable-after-conditioning neurons and among stable-after-conditioning and new neurons. The connectivity strength among stable-after-conditioning and new neurons was significantly lower than that among stable-after-conditioning neurons. In the green ensembles, we found that connectivity strength among lost neurons was significantly lower than that among stable-before-conditioning neurons and that among stable-before-conditioning and lost neurons (Figure 4J). The connectivity strength among stable-before-conditioning neurons was significantly larger than that among stable-after-conditioning neurons. Moreover, after conditioning, the connectivity strength among stable-after-conditioning neurons was significantly higher than that among stable-after-conditioning and new neurons and that among new neurons.

In summary, poor functional connectivity was observed in lost neurons (in comparison with stable neurons) in both blue and green ensembles. Stable neurons had either similar or weaker connectivity after conditioning in both blue and green ensembles. The new neurons, however, exhibited different local connectivity properties between blue and green ensembles. To be more specific, new neurons in blue ensembles exhibited not only similar relative degree and connectivity strength as the stable-after-conditioning neurons, whereas new neurons in green ensembles show larger relative degree and smaller connectivity strength than stable-after-conditioning neurons. In addition, new neurons in blue ensembles demonstrated a significantly larger clustering coefficient and intra-subgroup connectivity strength compared to stable-after-conditioning neurons, whereas new neurons in green ensembles showed a similar clustering coefficient and weaker intra-subgroup connectivity strength compared to stable-after-conditioning neurons. These results indicated that new neurons in the blue (conditioned) ensembles may play an essential role in learning.

### 3.5. Conditioning enhanced the connectivity strength between new neurons and all recorded neurons in blue ensembles

**Figure 5:**
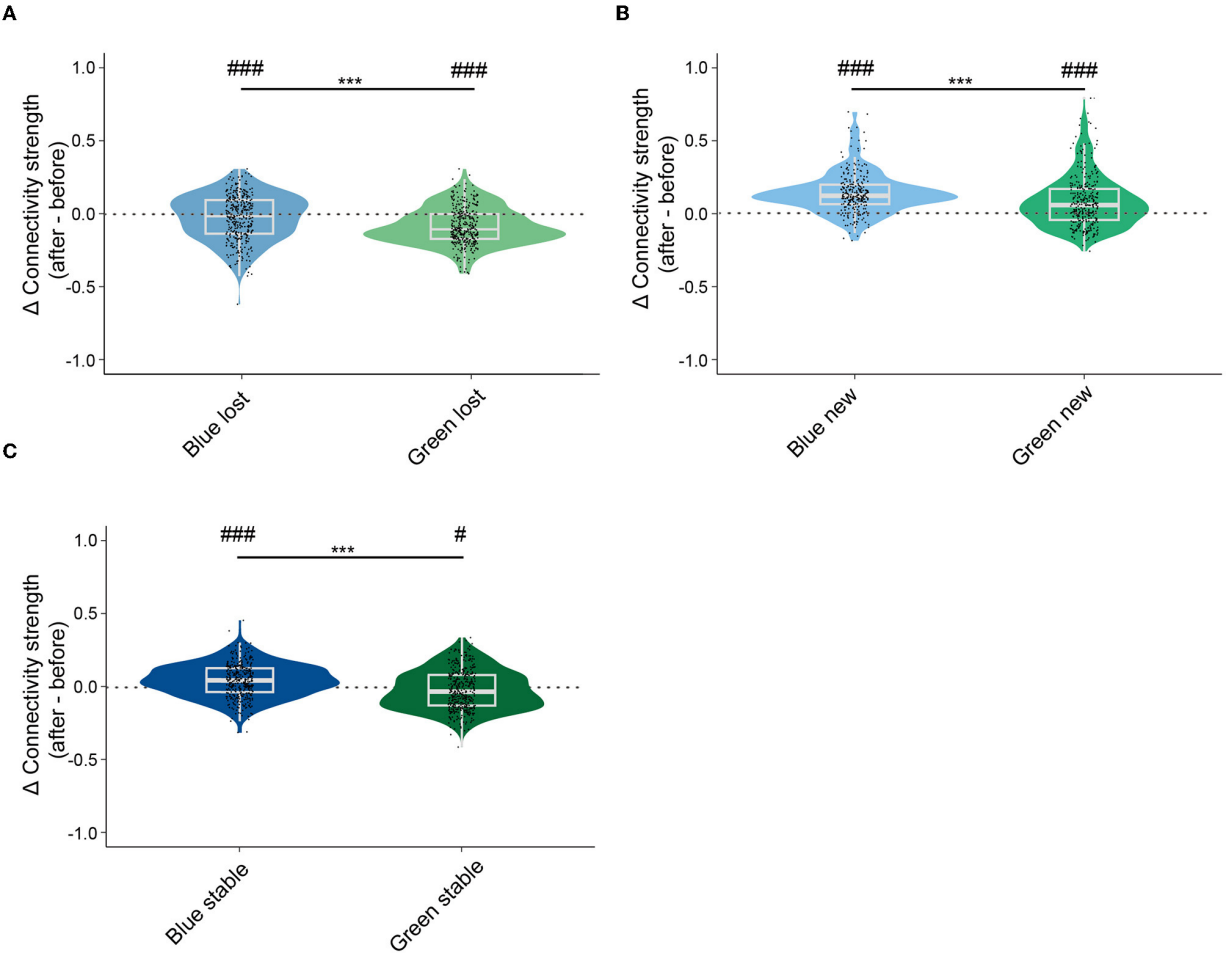
Lost neurons lost, new neurons gained, and stable neurons in green ensembles decreased more than stable neurons in blue ensembles increased in connectivity strength of ensembles. **(A)** Changes in global connectivity strength of lost neurons before and after conditioning. Lost neurons in blue ensembles: −0.027 ± 0.008, *n* = 370; lost neurons in green ensembles: −0.087 ± 0.007, *n* = 366. One sample *t*-test, *p* < 0.001 in lost neurons in blue ensembles; one sample *t*-test, *p* < 0.001 in lost neurons in green ensembles; Mann–Whitney test, *p* < 0.001 in lost neurons in blue ensembles vs. lost neurons in green ensembles. **(B)** Changes in global connectivity strength of new neurons before and after conditioning. New neurons in blue ensembles: 0.141 ± 0.008, n =274; new neurons in green ensembles: 0.081 ± 0.010, *n* = 331. One sample *t*-test, *p* < 0.001 in new neurons in blue ensembles; one sample *t*-test, *p* < 0.001 in new neurons in green ensembles; Mann–Whitney test, *p* = 0.001 in new neurons in blue ensembles vs. new neurons in green ensembles. **(C)** Changes in global connectivity strength of stable neurons before and after conditioning. Stable neurons in blue ensembles: 0.045 ± 0.006, *n* = 386, stable neurons in green ensembles: −0.016 ± 0.006, *n* = 425. One sample *t*-test, *p* < 0.001 in stable neurons in blue ensembles; one sample *t*-test, *p* = 0.016 in stable neurons in green ensembles; Mann–Whitney test, *p* < 0.001 in stable neurons in blue ensembles vs. stable neurons in green ensembles. The median, quarterlies, and 95% of confidence interval are illustrated in the box charts. ^#^represents the significant level compared to zeros ^#^*p* < 0.05, ^##^*p* < 0.01, ^###^*p* < 0.001, and ***p < 0.001.

To capture the change in connectivity in all recorded neurons, we analyzed the connectivity strength between stable, lost, new neurons, and all neurons in the recording site before and after conditioning. Lost neurons in both blue and ensembles had smaller connectivity strength with all neurons after conditioning (Figure 5A). Lost neurons in the green ensembles had a significantly larger change in connectivity strength with all neurons compared to those in blue ensembles. New neurons in both blue and green ensembles had significantly larger connectivity strength with all neurons after conditioning (Figure 5B). New neurons in blue ensembles had larger connectivity strength with all neurons than that in green ensembles. Interestingly, for stable neurons, a significant increase in connectivity strength with all neurons after conditioning was observed in the blue ensembles (Figure 5C). Conversely, a significant decrease in connectivity strength in stable neurons with all neurons after conditioning was observed in the green ensembles. Hence, the relative connectivity strength of stable neurons in green ensembles decreased significantly more than the stable neuron in the blue ensembles. Compared to the results within the ensembles, connectivity strength calculated in all neurons exhibited a similar trend to those calculated in ensembles.

## 4. Discussion

In our study, after visual-cued fear conditioning, we found that the proportion of neurons that responded only to blue (conditioned) light was significantly increased. However, after defining the ensembles based on functional connectivity, we found no significant difference in local and global connectivity properties except a significant enhancement of connectivity strength in the blue ensembles after conditioning. By classifying subgroups of neurons in the ensembles, we observed a differentiated functional shift within the blue and green ensembles, respectively. For stable neurons, a significant decline in the clustering coefficient was only observed in the blue ensembles. At the same time, a significant decrease in connectivity strength was only found in the green ensembles. For lost neurons, significantly weaker performances were illustrated in all parameters compared with stable-before-conditioning neurons in blue ensembles, but lost neurons in green ensembles exhibited significant differences compared with stable-before-conditioning neurons only excluding the clustering coefficient. Most importantly, we found that neurons newly recruited to the blue ensembles demonstrated similar performance in relative degree and connectivity strength and a higher clustering coefficient compared to the stable-after-conditioning neurons. Otherwise, new neurons in green ensembles showed a higher relative degree but a lower connectivity strength compared to the stable-after-conditioning neurons, whereas a similar performance in clustering coefficient. Moreover, not only new neurons in blue ensembles underwent an enhancement of connectivity strength throughout the whole population after conditioning, but a significant increase in connectivity strength improvement compared to green ensembles was also observed. These results implied that new neurons in the conditioned ensembles may play an essential role in memory formation.

Since the research of Wiesel and Hubel in the 1960s, the plasticity and stability of visual cortex neurons have been the subject of continuous investigation (Wiesel and Hubel, 1963; Mrsic-Flogel et al., 2007; Wandell and Smirnakis, 2009; Hengen et al., 2013; Lütcke et al., 2013; Clopath et al., 2017). Recent experiments have demonstrated that less than half of the neurons remain active in recordings up to 46 days under two-photon calcium imaging (Pérez-Ortega et al., 2021). In our study, changes in the identity of neurons in the ensembles after conditioning were also observed. We found that 46.3 ± 5.7% of neurons in the blue ensembles and 38.9 ± 6.5% of neurons in the green ensembles were lost (Figure 4B). Consistently, we also found that the connectivity strength of the lost neurons was significantly lower than that of the stable neurons in both blue and green ensembles (Figures 4E, H). One of the functions of ensembles is to maintain a stable state while continuously replacing individual neurons (Mrsic-Flogel et al., 2007; Hengen et al., 2013; Lütcke et al., 2013; Clopath et al., 2017). Based on the previous finding that the participation of neurons in the ensembles may depend on the stability of their dendritic spines (Yuste and Bonhoeffer, 2001), we suspected that the connectivity strength may be correlated with the strength of spines. Specifically speaking, transient spines in the lost neurons were thin and cause the absence of neurons as time passed, while stable neurons' persistent spines were consistently thick and maintained their existence across the recording period (Holtmaat et al., 2005). Moreover, there was a significantly lower percentage of blue new neurons compared to blue stable-after-conditioning neurons (Figure 4B) and no significant difference in the green ensembles, which may indicate that learning changed the natural turnover of different types of neurons. Together with the findings before, we believe that the flexibility of ensembles may help with memory acquisition and the stability may contribute to memory retention.

Several studies have demonstrated that after experiencing associative learning, layer 2/3 neurons in V1 performed better for task-related stimuli (Poort et al., 2015; Jurjut et al., 2017; Pakan et al., 2018). In our experiments, we also found that the proportion of V1 neurons responding to blue light (conditioned light) was significantly increased after fear conditioning (Figure 1I). On the contrary, the proportion of V1 neurons responding to green light (control light) did not change significantly (Figure 1I). In addition, the blue ensembles gained a significant improvement in connectivity strength after conditioning but not green ensembles (Figure 3C). This implies that the mouse primary visual cortex is capable of undergoing plastic changes not only in response to grating orientation (Henschke et al., 2020) but also to color stimuli. It is worth noting that the trend in the number of neurons that respond exclusively to one type of light may not follow a similar pattern in the case of neuronal ensembles. This discrepancy may arise from variations in the definitions of neurons belonging to ensembles vs. those that are considered responsive. Hence, further research is necessary to investigate this topic in future.

According to Hebb's theory, “neurons that fire together wire together,” which means that when a group of neurons was repeatedly activated together, they form a memory trace through increased connectivity at their synapses (Hebb, 2005). Therefore, the enhancement of functional connectivity of neurons on average in learning-related ensembles may be caused by the learning-related plasticity change, for example, their connections in synapses. This implication was also supported by the significant rise of connectivity strength in blue stable (Figure 5C) and blue new (Figure 5B) neurons from which formed learning-related ensembles together.

More importantly, new neurons that were newly recruited to the learning-related ensembles also played an essential role in memory formation. Similar to the recent findings that silent synapses could become unsilent after Hebbian pairing, recruiting new active connections into a neuron's input matrix (Vardalaki et al., 2022), new neurons in the current study were also “silent” and shifted into “unsilent” neurons after learning. They performed not only a powerful connectivity strength and a noticeable connection within the ensembles (Figure 4E) but also a significantly more densely clustering coefficient and significantly larger intra-subgroup connectivity strength than stable-after neurons (Figure 4D), that is to say, new neurons wired extensively and strongly and connected more densely with their neighbors compared to the stable-after neurons in learning-related ensembles. Therefore, we suppose that there is a more centralized role of the new neurons, and they may play in promoting information transmission in the associative learning process.

One limitation of this study is that we were unable to directly distinguish neuron types since GCaMP6s labels all neurons and there is no current method to differentiate different types of neurons. GABAergic interneurons are also very important in the neural network-level regulation. For example, parvalbumin (PV) interneurons could stabilize the cortical circuit while somatostatin (SOM) interneurons could modulate the gain of pyramidal neurons (Bos et al., 2020; Millman et al., 2020). Knowing neuronal types will provide more information, and we might consider such in future by injecting AAV1-Syn-Flex-GCaMP6f into various transgenic mice (e.g., PV-Cre, SOM-Cre, and VIP-Cre) (Lee et al., 2022).

## Data availability statement

The original contributions presented in the study are included in the article/supplementary material, further inquiries can be directed to the corresponding author.

## Ethics statement

The animal study was reviewed and approved by Animal Ethics Committee of School of Basic Medical Sciences at Fudan University.

## Author contributions

J-YZ and BY conceived the study and polished the manuscript. Y-GS performed the two-photon calcium imaging and fear conditioning experiments of mice. X-DC performed the histochemistry experiments. Y-GS and W-XS analyzed the data and wrote the manuscript. Y-GS and W-XS constructed figures with Z-YL. All authors discussed the results and commented on the manuscript.
